# A Robust *H*_*∞*_ Controller for an UAV Flight Control System

**DOI:** 10.1155/2015/403236

**Published:** 2015-06-09

**Authors:** J. López, R. Dormido, S. Dormido, J. P. Gómez

**Affiliations:** ^1^Dynamic Systems Research Group, Universidad Politécnica de Madrid (ETSIA/EUITA), Plaza Cardenal Cisneros 3, 28040 Madrid, Spain; ^2^Department of Computer Sciences and Automatic Control, UNED, Calle Juan del Rosal 16, 28040 Madrid, Spain

## Abstract

The objective of this paper is the implementation and validation of a robust *H*_*∞*_ controller for an UAV to track all types of manoeuvres in the presence of noisy environment. A robust inner-outer loop strategy is implemented. To design the *H*_*∞*_ robust controller in the inner loop, *H*_*∞*_ control methodology is used. The two controllers that conform the outer loop are designed using the *H*_*∞*_ Loop Shaping technique. The reference vector used in the control architecture formed by vertical velocity, true airspeed, and heading angle, suggests a nontraditional way to pilot the aircraft. The simulation results show that the proposed control scheme works well despite the presence of noise and uncertainties, so the control system satisfies the requirements.

## 1. Introduction

There is a considerable and great interest in using unmanned air vehicles (UAVs) to perform a multitude of tasks [1]. UAVs are gaining more powerful skills to accomplish a wide range of missions with high efficiency and high accuracy rate. They are becoming vital warfare and homeland security platforms because they significantly reduce both the costs and the risk to human life and first-responder capabilities. UAVs have many typical applications such as intervention in industrial plants, natural disasters intervention, cooperation with other ground robots in demining operations, through aerial mapping, remote environmental research, pollution assessment and monitoring, fire-fighting management, security, for example, border monitoring, law enforcement, scientific missions, agricultural and fisheries applications, oceanography, or communications relays for wideband applications. Due to their numerous benefits, it would be nice to decrease the global cost of this type of aircraft. In this sense, the flight control design problem for low cost UAV still requires significant efforts, being the control and dynamic modeling of UAVs which is an attractive field of research.

The control of UAVs is not an easy task as the UAV is a multi-input multioutput (MIMO), under actuated, unstable, and highly coupled system. Many traditional control strategies have been used over the years for the control of UAVs, such as linear quadratic regulator (LQR) [2, 3].

Robust techniques have also been applied to design controllers to achieve robust performance and simultaneously guarantee stability when system deviates from its nominal design condition and/or is subjected to exogenous disturbances. In particular, robust *H*_*∞*_ control method by Zames [4, 5] has been used in flight control systems for both lateral and longitudinal dynamics of aircraft [6–8].

In this work, an inner-outer loop control architecture applied to the longitudinal and lateral flight motions is implemented using the *H*_*∞*_ Loop Shaping Design procedure [9, 10] to synthesize the inner-loop controller. The technique decouples the longitudinal and lateral dynamics and minimizes the cross effects involved. The feasibility of the controller is analyzed.

The control scheme is implemented on a 6-DOF nonlinear simulation model. Different simulation results are presented to show the robustness of the proposed control architecture. The paper is structured as follows.  Section 2 presents the aircraft model and its linearization.  Section 3 describes the control problem, presenting the control objectives and the control scheme. Design results are analyzed in Section 4. Flight test results are presented in Section 5.

## 2. Aircraft Model

### 2.1. Fully Nonlinear Dynamic Model

**Figure 1:**
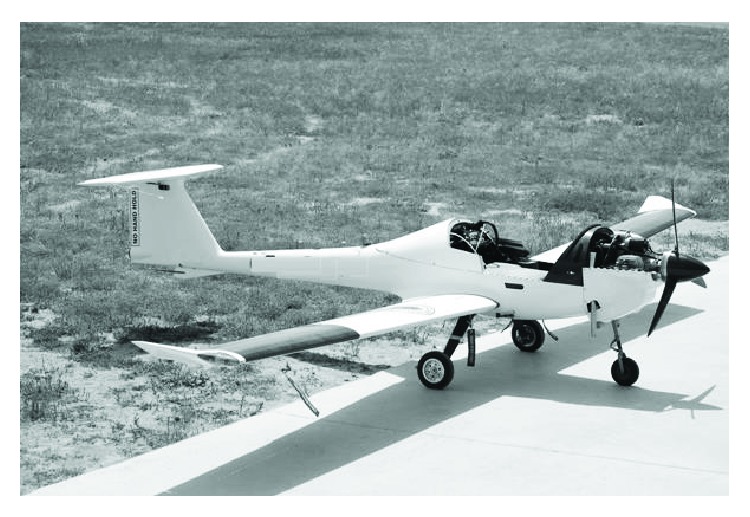
KUAV scale model.

The UAV is a 1/3 scaled down model of a Diamond Katana DA-20 shown in Figure 1.

The main characteristics of the aircraft are as follows:

- span 3.9 m,
- wing surface 1.47 square meters,
- mean aerodynamic chord 0.39 m,
- mass 18–30 kg,
- cruise velocity 130 km/h,
- maximum velocity 200 km/h,
- engine power 8 HP,
- centre of gravity between 15 and 31% of mean aerodynamic chord.

Aircraft dynamics is described as a full 6-degree-of-freedom (DOF) 13-state high fidelity UAV nonlinear model. The nonlinear model has been developed in standard body axes centered at the aircraft center of gravity where *x* points forward, through the aircraft noise, *y* is directed to the starboard (right), and *z* is directed through the belly of the aircraft.

Using the notation given by Stevens and Lewis [11], the flight dynamic model that describes the rigid body motion of the aircraft is given by the following equations.

Force equations are as follows:

(1) $$\begin{array}{c} \dot{U}=RV-QW+g_{x}+\frac{F_{x}}{m},\\ \dot{V}=-RU+PW+g_{y}+\frac{F_{y}}{m},\\ \dot{W}=QU-PW+g_{z}+\frac{F_{z}}{m}. \end{array}$$

Moment equations are as follows:

(2) $$\begin{array}{c} \left[\begin{array}{c} \dot{P}\\ \dot{Q}\\ \dot{R} \end{array}\right]=J^{-1}\left[\begin{array}{c} L\\ M\\ N \end{array}\right]-\left[\begin{array}{ccc} 0 & -R & Q\\ R & 0 & -P\\ -Q & P & 0 \end{array}\right]J\left[\begin{array}{c} P\\ Q\\ R \end{array}\right]. \end{array}$$

Kinematic equations are as follows:

(3) $$\begin{array}{c} \dot{\phi }=P+\tan\theta \left(Q\sin\phi +R\cos\phi \right),\\ \dot{\theta }=Q\cos\phi -R\sin\phi ,\\ \dot{\psi }=\frac{\left(Q\sin\phi +R\cos\phi \right)}{\cos\theta }. \end{array}$$

Navigation equations are as follows:

(4) $$\begin{array}{c} \left[\begin{array}{c} {\dot{p}}_{N}\\ {\dot{p}}_{E}\\ -\dot{h} \end{array}\right]=B^{-1}\left[\begin{array}{c} U\\ V\\ W \end{array}\right], \end{array}$$

where *m* is the mass; (*U*, *V*, *W*) are the body axis velocity states; (*P*, *Q*, *R*) are the body axis rates; *ϕ*, *θ*, *ψ* are the roll, pitch, and yaw angles, respectively; and (*p*_*N*_, *p*_*E*_, *h*) are the north, east, and height positions. *F* = (*F*_*x*_, *F*_*y*_, *F*_*z*_) represents the aerodynamic force vector and *M* = (*L*, *M*, *N*) represents the moment vectors. *J* is the aircraft inertia:

(5) $$\begin{array}{c} J=\left[\begin{array}{ccc} J_{XX} & 0 & -J_{xz}\\ 0 & J_{yy} & 0\\ -J_{xz} & 0 & J_{zz} \end{array}\right]; \end{array}$$

**B** is the inertial top body transformation matrix; (*g*_*x*_, *g*_*y*_, *g*_*z*_) is the gravity vector, which is the transformation of the (0,0, *g*) NED-frame gravity vector to the body axis frame, as shown below:

(6) $$\begin{array}{c} \left[\begin{array}{c} g_{x}\\ g_{y}\\ g_{z} \end{array}\right]=B\left[\begin{array}{c} 0\\ 0\\ g \end{array}\right], \end{array}$$

where
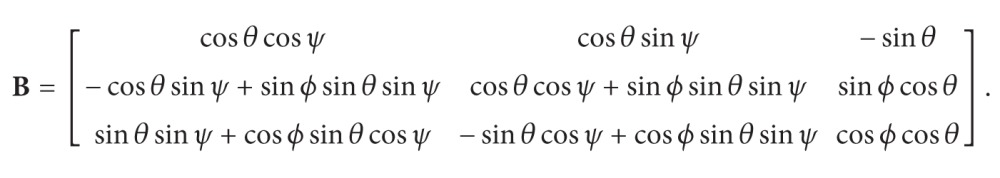
(7)


The resulting model is described by a thirteen-state order model [12]. Due to the complexity and the uncertainty inherent to aerodynamic systems, the dynamic model was identified by a complete identification flight set through the full envelope. See Stevens and Lewis for details [11].

### 2.2. Linearized Dynamic Model

The nonlinear dynamic model described in Section 2.1. is linearized about certain trimmed operating conditions. This process is accomplished by perturbing the state and control variables from steady state.

The mathematical formulation of the dynamic system is modeled with standard continuous time invariant state space formulation given by (8). Where *A* is a 13 × 13 matrix, *B* a 13 × 4 matrix, *C* a 12 × 13 matrix, and *D* is a 12 × 4 matrix,

(8) $$\begin{array}{c} \dot{x}=Ax+Bu\\ y=Cx+Du, \end{array}$$

and the state, output, and control vectors are, respectively,

(9) $$\begin{array}{c} x={\left[\begin{array}{ccccccccccccc} V_{T} & \alpha & \beta & \phi & \theta & \psi & P & Q & R & p_{N} & p_{E} & h & \text{pow} \end{array}\right]}^{T},\\ y={\left[\begin{array}{cccccccccccc} a_{x} & a_{y} & a_{z} & P & Q & R & \text{lon} & \text{lat} & h & {\dot{p}}_{N} & {\dot{p}}_{E} & \dot{h} \end{array}\right]}^{T},\\ u={\left[\begin{array}{cccc} \delta_{tl} & \delta_{e} & \delta_{a} & \delta_{r} \end{array}\right]}^{T}. \end{array}$$

The state vector (*x*) components are true airspeed (*V*_*T*_), angle of attack (*α*), sideslip angle (*β*), roll angle (*ϕ*), pitch angle (*θ*), yaw angle (*ψ*), roll rate (*P*), pitch rate (*Q*), yaw rate (*R*), north position (*p*_*N*_), east position (*p*_*E*_), altitude (*h*), and power (pow).

The output vector (*y*) is formed by *x*-component of acceleration (*a*_*x*_), *y*-component of acceleration (*a*_*y*_), *z*-component of acceleration (*a*_*z*_), roll rate (*P*), pitch rate (*Q*), yaw rate (*R*), longitude (lon), latitude (lat), altitude (*h*), north position derivative, east position derivative, and altitude derivative.

The control vector (*u*) is defined by throttle (*δ*_*tl*_), elevator (*δ*_*e*_), aileron (*δ*_*a*_), and rudder (*δ*_*r*_).

The dynamics are linearized about a representative flight condition. This nominal condition is *V*_*T*_ = 30 ms^−1^, centre of gravity position equal to 25% of mean aerodynamic chord, *ϕ* = 0 rad, *ψ* = 0 rad, *R* = 0 rad, *P* = 0 rad, *θ* = 0 rad, rate of climb = 0 rad, and lateral acceleration = 0 rad.

## 3. Control Technique

### 3.1. Control Objectives

The main objective is the design of a robust controller to track all types of input commands in a noisy environment. The controller has to be designed as a trade-off robustness and performance in order to fulfill the specifications described in this section.

#### 3.1.1. Closed Loop Specifications

Stability of the aircraft, minimal overshoot, and reasonably long settling time are important constraints in the design. Translated into physical design goals, the controller must perform the following specifications:

- altitude response: overshoot < 5%, rise time < 5 s, and settling time < 20 s,
- heading angle response: overshoot < 5%, rise time < 3 s, and settling time < 10 s,
- flight path angle response: overshoot < 5%, rise time < 1 s, and settling time < 5 s,
- airspeed response: overshoot < 5%, rise time < 3 s, and settling time < 10 s,
- cross coupling between airspeed and altitude: for a step in commanded altitude of 30 m, the peak value of the transient of the absolute error between airspeed and commanded airspeed should be smaller than 0.5 ms^−1^; conversely, for a step in commanded airspeed of 2 ms^−1^, the peak value of the transient of the absolute error between altitude and commanded altitude should be smaller than 5 m.

#### 3.1.2. Gust Rejection

Second objective of the control system is to include robustness to gust effects on the aircraft. In this sense, turbulence can be considered as a stochastic process defined by its velocity spectra. For an aircraft flying at a cruise speed *U*, a commonly used velocity spectra for turbulence model is the Dryden spectra [13]:

(10) $$\begin{array}{c} \Phi_{v}=\frac{2L_{v}\sigma^{2}\left(1+12{\left(L_{v}/U\right)}^{2}w^{2}\right)}{\pi U{\left(1+4{\left(L_{v}/U\right)}^{2}w^{2}\right)}^{2}}, \end{array}$$

where *w* is the frequency in rad s^−1^, *σ* is the turbulence standard deviation, and *L*_*v*_ is the turbulence scale length. The turbulence parameters values for severe gust conditions are given by [14]

(11) $$\begin{array}{c} \sigma =0.1+0.00733h,\;300<h<600\;\text{m}\\ \sigma =3.04+0.00244h,\;600<h<1400\;\text{m}\\ \sigma =6.45\;\text{m}/\text{s},\;1400<h<5800\;\text{m}\\ \sigma =8.40-0.000336h,\;h>5800\;\text{m}\\ L_{v}=\frac{h}{{\left(0.177+0.00274h\right)}^{1.2}}, \end{array}$$

where *h* is the altitude. Our gust rejection specification is to reject all disturbances below 13 rad s^−1^.

#### 3.1.3. Noise Rejection

Basically, the measured variables for the lateral control are the lateral acceleration and the yaw and roll rates measured in body fixed axis. For the selected sensors, the noise is high and concentrated in the frequency range above 30 rad s^−1^. Thus, high frequency specification is that in which all noise spectra, which normally occur above 30 rad s^−1^, should be rejected.

#### 3.1.4. Robustness Specifications

The controller designed has to be robust against uncertainty in the plant model. The robust specifications are defined as follows.

- Centre of gravity variation is as follows: stability and sufficient performance should be maintained for horizontal centre of gravity variations between 15% and 31% cbar (mean aerodynamic chord).
- Vertical centre of gravity must not suffer variations: it should remain at 0% cbar.
- Mass variations are as follows: stability and sufficient performance should be maintained for aircraft mass variations between 18 and 30 kg.
- Time delay is as follows: stability and sufficient performance should be maintained for transport delays from 0 to 60 ms.
- Speed variations are as follows: stability and sufficient performance should be maintained for speed variations from 1.23VS (stall velocity) to 55 m s^−1^ (200 Km/h).

### 3.2. Controller Design

**Figure 2:**
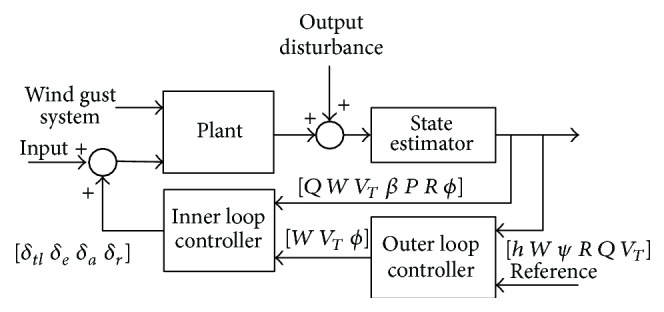
Controller architecture.

The control architecture is based on that proposed by Tucker and Walker [13]. As Figure 2 shows, basically, it consists of two loops: an inner-loop controller to achieve stability and robustness to expected parameter uncertainty and an outer loop for tracking reference performances.

The design of the inner loop is focused on maintaining the vertical velocity deviation, the heading angle deviation, and the airspeed deviation near zero.

Two different controllers conform to the outer loop: the altitude controller and the heading angle-lateral deviation controller. Both controllers are synthesized using the *H*_*∞*_ Loop Shaping technique (see [9, 15, 16]).

**Figure 3:**
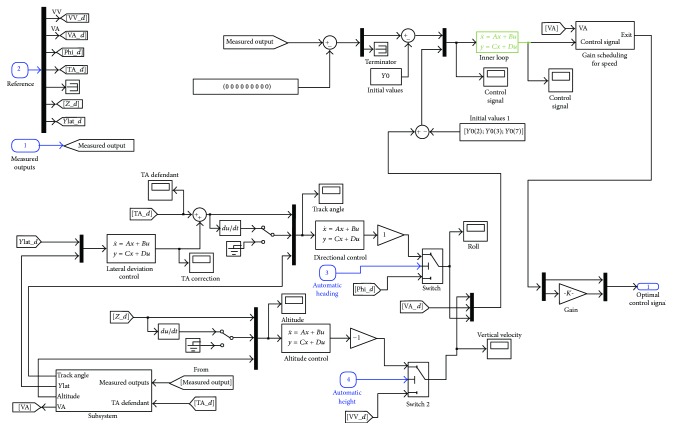
Framework to design the *H*_*∞*_ controller.

Figure 3 shows the general framework used in the design process.

#### 3.2.1. The Inner Loop Synthesis Procedure

**Figure 4:**
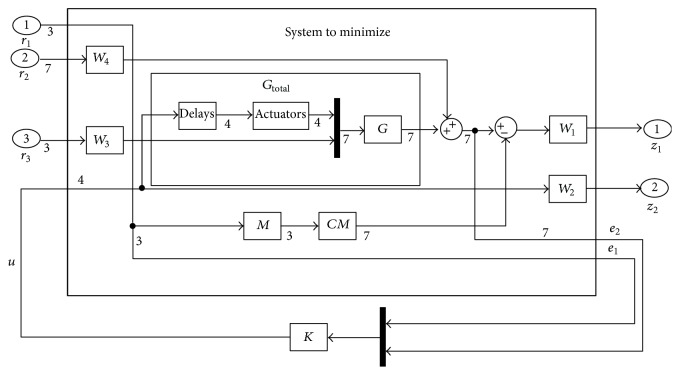
Inner loop architecture.

Figure 4 shows the inner loop architecture. Its main goal is to minimize both the deviation to desired output and the control effort. *r*_*i*_ ∈ *R* ^3^ is the reference input vector, whose components are the vertical speed, airspeed, and the roll angle. *u* ∈ *R* ^4^ is the control signal. *z*_1_ ∈ *R* ^3^ is the vector of performance outputs. *z*_2_ ∈ *R* ^2^ is the vector of weighted control inputs. The feedback variables are the vertical speed, airspeed, the roll angle, the pitch rate, the yaw rate, the roll rate, and the sideslip.

The total plant *G*_total_ is formed by the plant *G* (the linearized UAV model), the actuators model, and the corresponding delays. These delays are modelized using the first order Pade approximations. They are used to represent plant uncertainties in the high frequency range such as modeling errors and neglected actuator dynamics. Four delays of 100 ms are included in the plant model, one in each input including the throttle.

The actuator model for *δ*_*e*_, *δ*_*a*_, and *δ*_*r*_ is given by the first-order linear approximation 10/(*s* + 10) and the engine model is represented by 2/(*s* + 2).

The sensor noise is represented by means of white noise model. The standard deviations of the sensor noise corresponding to the output vector are 0.1 ms^−2^ for accelerations, 0.005 rad s^−1^ for angular velocity, 5 m for position, and 0.5 ms^−1^ for velocity.

The controller *K* is designed using the *H*_*∞*_ technique. It must guarantee the stability and follow an ideal model, the so called matching model (*M*). That is, the closed loop system output *y*1 is expected to match *y*_*m*_ ∈ *R* ^3^, the output of the ideal model *M*.

The matching model *M*, which defines the behaviour of the vertical speed, the true speed, and the heading angle, consists of the following three second-order systems:

(12) $$\begin{array}{c} M=\left[\begin{array}{ccc} \frac{4^{2}}{s^{2}+2\cdot 4s+4^{2}} & 0 & 0\\ 0 & \frac{1.5^{2}}{s^{2}+2\cdot 1.5s+1.5^{2}} & 0\\ 0 & 0 & \frac{2.25^{2}}{s^{2}+2\cdot 2.25s+2.25^{2}} \end{array}\right]. \end{array}$$

The matching model is selected to accomplish desired behaviour of the vertical speed, airspeed, and roll angle to achieve the closed loop specifications detailed in Section 3.1. The cross coupling terms are zero, thus, defining the requirement for closed loop system as decoupled.

Four weights *W*_*i*_ (*i* = 1,…, 4) are used in the inner loop to accomplish the frequency dependent specifications on performance and robustness. They are added to maximize disturbances rejection and to minimize wind gusts effects and sensor noises.

*W*_1_ is related with reference tracking. So, its elements are selected as low pass filters. The yaw rate and roll rate are selected as pass band filters.

*W*_2_ is devoted to minimize the control effort. This is why it is selected as a high pass filter, where its gain and bandwidth are chosen to allow low frequency control effort and to minimize high frequency control effort.

*W*_3_ and *W*_4_ are unity matrix. They weight turbulences and output disturbances, respectively.

The controller's synthesis is accomplished using an iterative procedure. First, the weights are selected; then the controller *K* is synthesized and finally the resulting system performances are analysed.

After this iterative process, the weights selected are the following:

(13) W1=diag32(s+1)s+2·3s+32,10500ss/0.001+1,    10500ss/0.001+1,5500ss/0.001+1,    72(s+1)s+2·7s+72,72(s+1)s+2·7s+72,    32(s+1)s+2·3s+328500ss/0.001+1,W2=diag0.5s/0.1+1s/0.008+1,0.5s/0.1+1s/0.008+1,    0.5s/0.1+1s/0.008+1,0.5s/0.1+1s/0.008+1,W3=I3,W4=I7.

After some iterations, a stabilizing controller *K*(*s*) is determined. This controller minimizes the variables *z*_1_ y *z*_2_ (see Figure 3) which corresponds to deviation between the desired output, provided by the matching model and the real aircraft output and control effort. The subresulting suboptimal robust stability margin is *γ* = 4.98.

#### 3.2.2. The Outer Loop Synthesis Procedure

**Figure 5:**
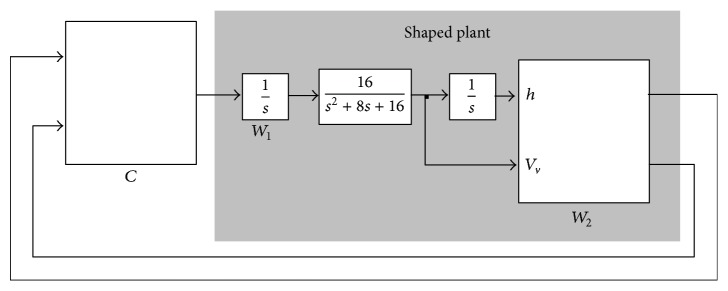
Outer-loop altitude command tracker.

**Figure 6:**
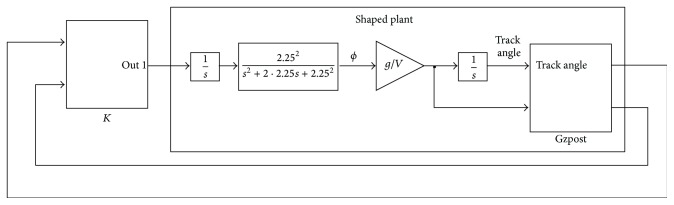
Outer-loop heading angle and lateral deviation controller.

Two different controllers conform to the outer loop: the altitude controller (see Figure 5) and the heading angle-lateral deviation controller (see Figure 6). The two outer-loop controllers are synthesized using the *H*_*∞*_ Loop Shaping technique [16].

Figure 5 shows the first problem to be solved, where *C* is the controller and *W*_1_ and *W*_2_ are the weights used to tune the optimization. The simplified models of the plant used to synthesize these controllers are those defined in the matching model.

In the design of the altitude controller, an output integrator is used to provide height and vertical velocity outputs. An input integrator is used to improve the low frequency behaviour.

In a similar way in the heading angle-lateral deviation controller design an output integrator is used to provide yaw angle and its derivative outputs. An input integrator is used to improve the low frequency behaviour.

The gamma values encountered are 3.18 and 2.5 for the altitude controller and the heading angle-lateral deviation controller, respectively.

The heading angle and lateral deviation controllers have been built together due to the hard interaction between the variables implied which motivates a tedious iterative process when individual controllers were designed. In this approach, these two controllers are synthesized jointly.

## 4. Design Results

The performance of a system can be represented by the sensitivity function *S*. The maximum singular value of *S* is an important boundary in this case. By using the largest singular value, we are effectively assessing the worst case scenario. Performance specification means the minimization of the sensitivity function as much as possible for low frequencies. At the same time, control effort should be small in the high frequency range.

**Figure 7:**
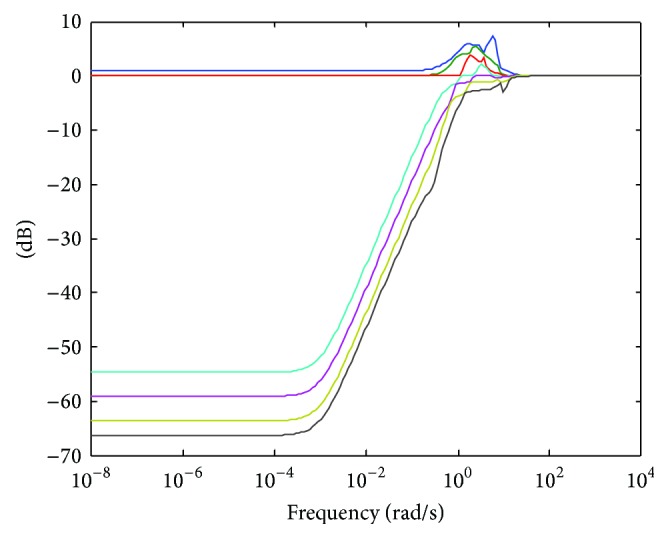
Singular values of the sensitivity function.

Figure 7 shows the sensitivity function. It is easy to see that our goal of minimising the sensitivity at low frequencies has been achieved. At high frequencies, the gain is unity and around the bandwidth there is a peak in the response.

This behaviour of the sensitivity enables good tracking reference at the low frequency range and noise reduction and robustness in the high frequency range.

**Figure 8:**
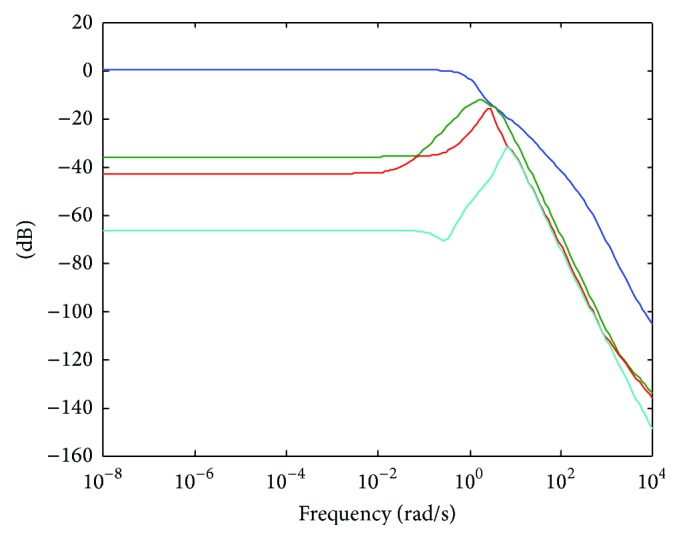
Singular values of the control effort.

Figure 8 shows the control effort behaviour which is lower in the high frequency range as it was expected.

Since the *H*_*∞*_ controller designed produces a 46-size state space realization, it is necessary to apply controller reduction techniques. A final state realization for the controller of dimension 27 is achieved using Hankel minimum degree approximation (MDA) without balancing reduction method [17].

**Figure 9:**
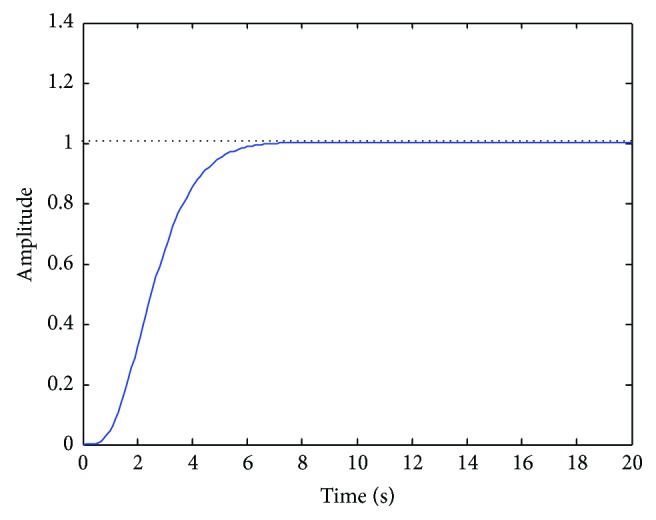
Lateral deviation step response (correct order reduction).

This method has been applied iteratively checking the frequency and time responses every step to evaluate the performance of the proposed UAV control scheme. One example of the time response in one step of this iterative process is shown in Figure 9.

**Figure 10:**
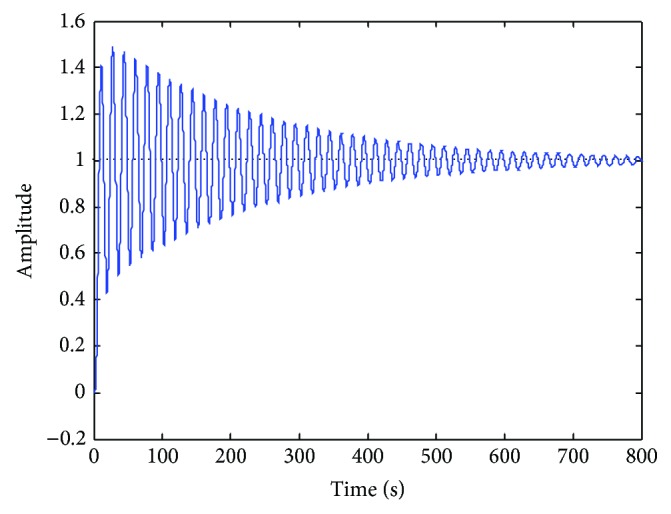
Lateral deviation step response (incorrect order reduction).

Figure 10 shows the effect of an incorrect order reduction. This performance is obtained when an order reduction is forced and the reduced controller is not able to maintain the desired specifications.

## 5. Simulation Tests Results

In order to validate the controller designed, a set of test cases have been developed. Below, an experience corresponding to 45-degree heading angle step response is shown. The results allow checking the performance of the aircraft in a noisy environment along this type of manoeuvre.

**Figure 11:**
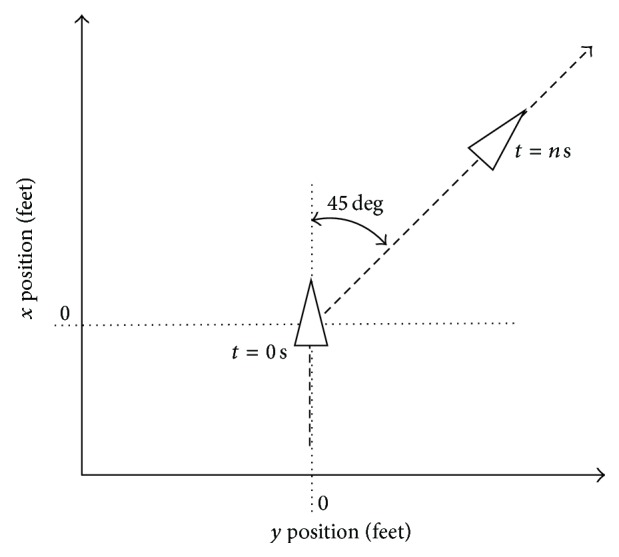
Airplane desired trajectory.

The airplane desired reference is illustrated in Figure 11. The dashed line is the desired trajectory.

**Figure 12:**
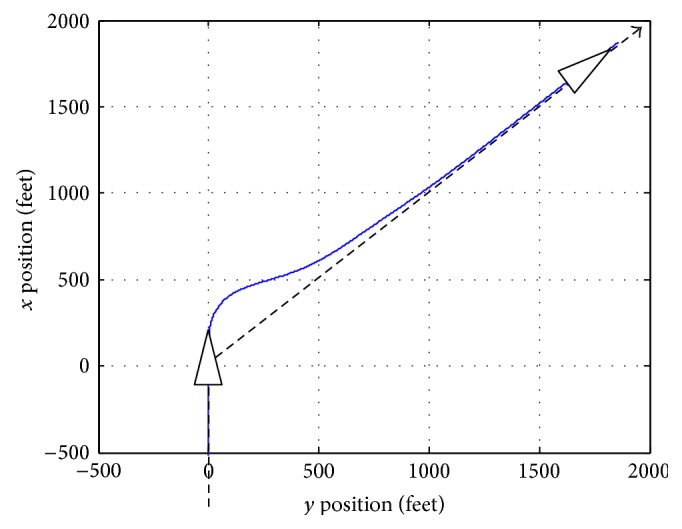
Airplane real trajectory.

Figure 12 shows the airplane simulated trajectory tracked. The dashed line is the desired trajectory and the continuous line is the real one.

**Figure 13:**
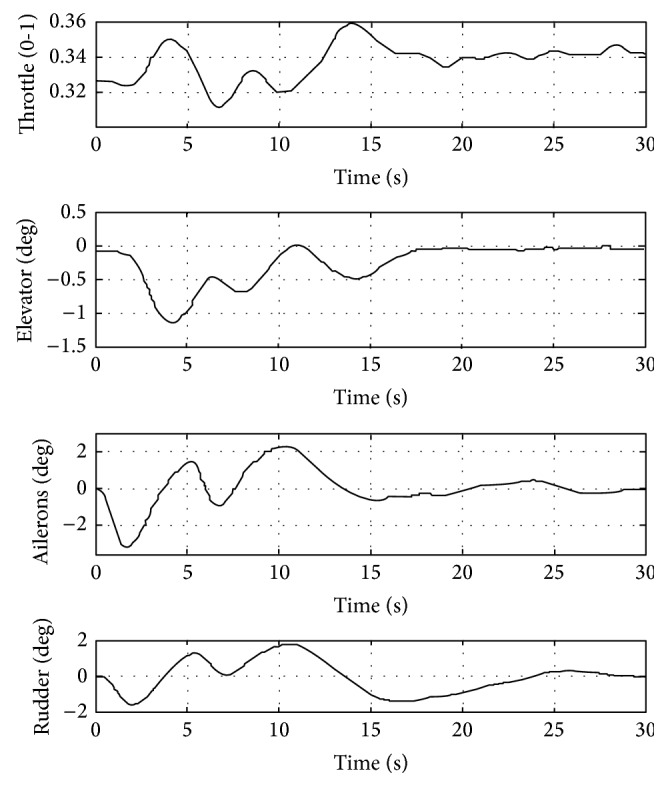
Control variables evolution during the 45-feet heading angle response.

The controller is able to manage adequately the output and to calculate the control vector. Control variables evolutions are shown in Figure 13. The throttle varies around 2% and elevator, ailerons, and rudder present a smooth behaviour. The aileron and rudder are deflected by the controller to order the 45-degree change of direction. Immediately, a sustentation loose typical in this type of manoeuvers is suffered by the aircraft. To compensate this trend, the elevator acts to raise the noise of the aircraft and slightly increase the throttle to maintain the velocity.

Figure 13 confirms that the control variables remain far from its saturation values. The power demand is less than 40% and the elevator, aileron, and rudder demanded deflections are less than 5 degrees. In this case, if the altitude holder is not connected, in 5 s, the airplane suffers an altitude loss of 3 m and rapidly it recovers the desired altitude, in about 5 s more. The UAV quickly corrects its heading angle turning to reduce the error. In about 4.5 s, the error is null; however, the airplane continues turning. This is produced because of the lateral deviation. If the airplane stops its turning movement in 4.5 s, it would continue straight ahead along a parallel line to the desired trajectory. To reduce the lateral deviation, it must continue turning and augmenting, in a first stage, the heading angle error. Following this strategy, the controller gains its tracking heading angle and its lateral deviation reduction goal.

## 6. Flight Test Results

For testing the whole system and the performance of the controller in flight, many real tests are accomplished. These tests are scheduled to validate in essence the physical design of the UAV, communications equipment, engine capabilities, and onboard software. A very important part of onboard software is the flight control system.

**Figure 14:**
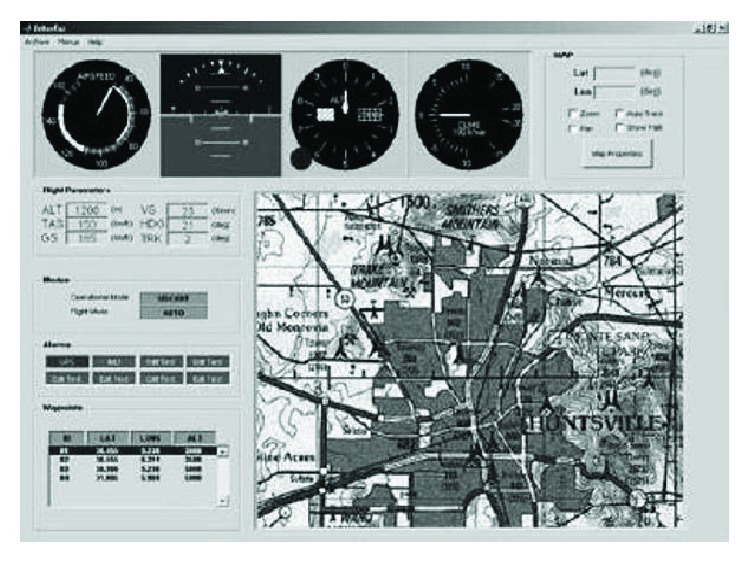
Ground Control Station GUI.

To manage the UAV platform, a ground station is developed (see Figure 14). It enables following the position and the attitude of the aircraft directly on a map shown in the computer. It also allows showing the main variables of the UAV which are sent through a radio link. The ground station allows introducing a set of waypoints. The autopilot takes care of both navigation and stability of the plane. The mission is planned via waypoints, placing on a geo referred map the position of each waypoint at the beginning of the mission. This mission can be easily modified during its execution by adding/changing/removing waypoints in the map.

The system provides a user-friendly interface used to display the plane position in real time on a map during the mission and to monitor some UAV parameters such as battery levels, speed, position and orientation, or the sensors measurements. The system also provides a radio link which allows a continuous exchange of data between the plane and the control station.

In an emergency case, the aircraft can switch to a PIL (pilot in the loop) mode in which the plane can be teleoperated from the control station by using a control-stick while the onboard autopilot remains on sleep mode.

**Figure 15:**
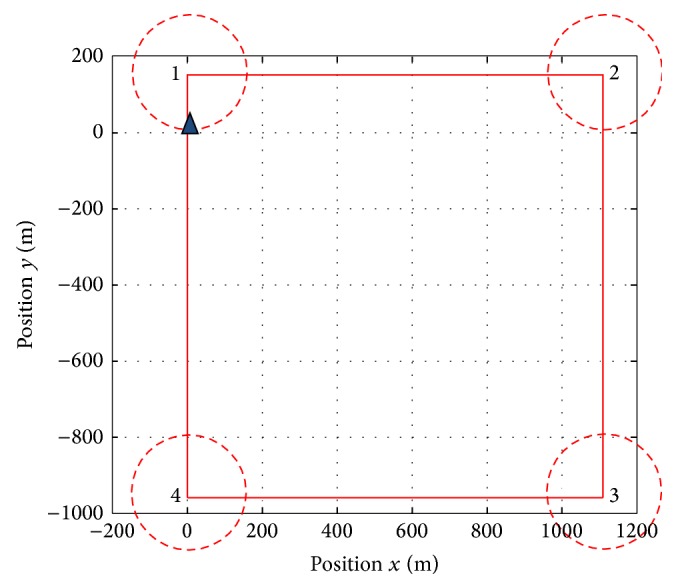
Waypoints and condition goals.

The selected test to illustrate the aptitudes of the autopilot designed is a circuit formed by four waypoints which is shown in Figure 15. The tracking reference trajectory is shaped for the waypoints labelled from one to four. The circles around the waypoint determine the instance when the reference input changes to the next waypoint (goal condition).

**Figure 16:**
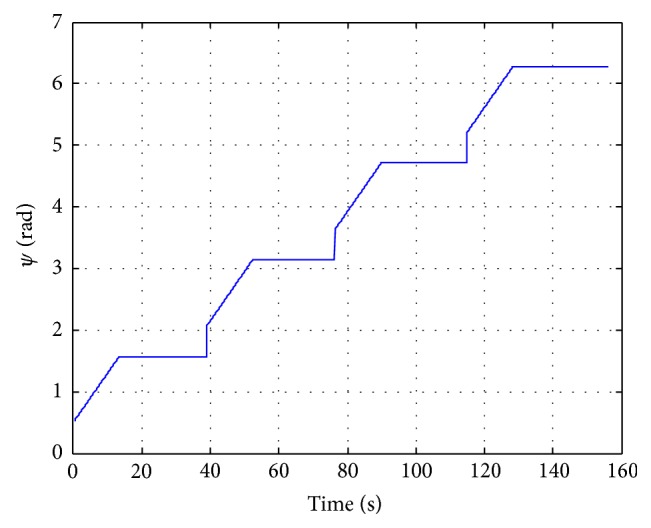
Smooth psi angle reference.

The reference is provided to the autopilot as a psi angle function and is built in a soft way using a combination of a step and a ramp. The reference is shown in the Figure 16.

**Figure 17:**
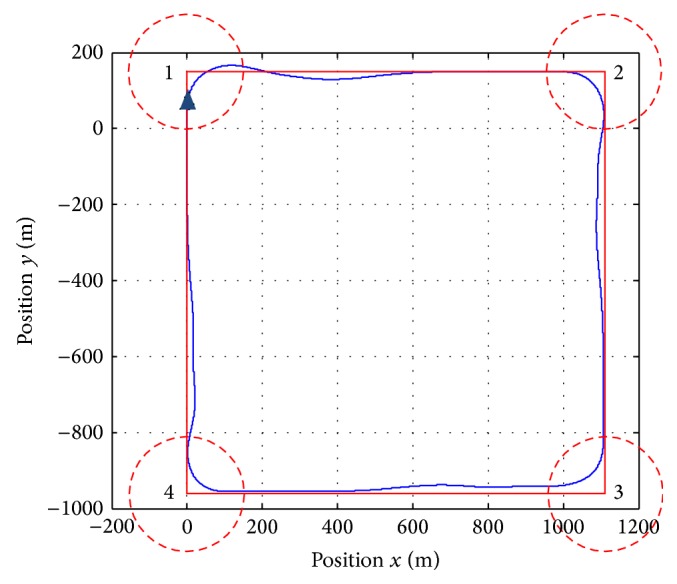
Real trajectory followed by the UAV.

Figure 17 shows how the UAV is capable of managing adequately the uncertainties and disturbances introduced by the modelling inaccuracies and the noisy output provided by the sensor. The response of the aircraft is not oscillating and it reaches the correct trajectory quickly when covering 600 m approximately which means 20 s at 30 ms^−1^ of mean velocity.

The entire trajectory covered is around 160 s and the psi angle and lateral deviation error are minimized satisfactorily. A desired decoupling between lateral and longitudinal dynamics is achieved.

**Figure 18:**
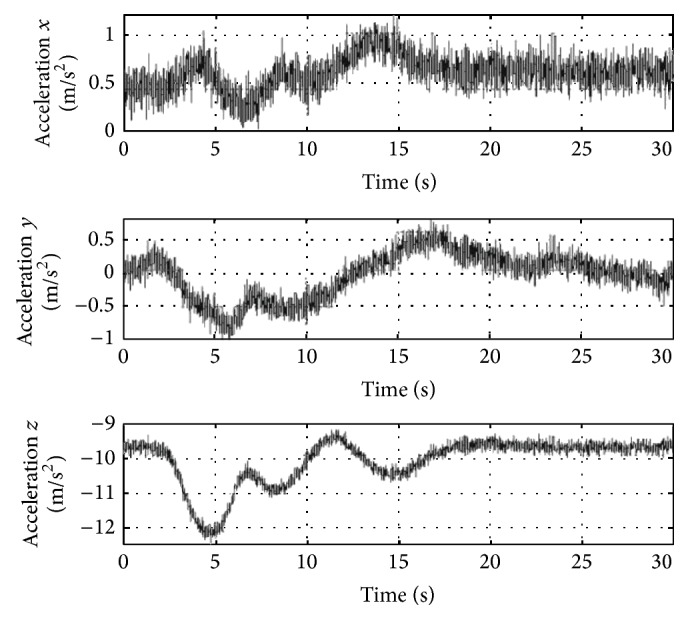
Accelerations measured.

Figure 18 shows the noisy accelerations output provided by the inertial sensors to the controller.

**Figure 19:**
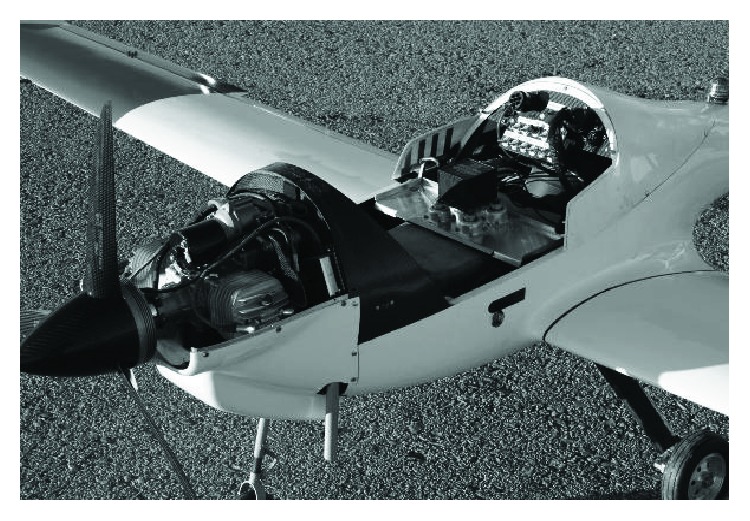
On-board HW equipment.

**Figure 20:**
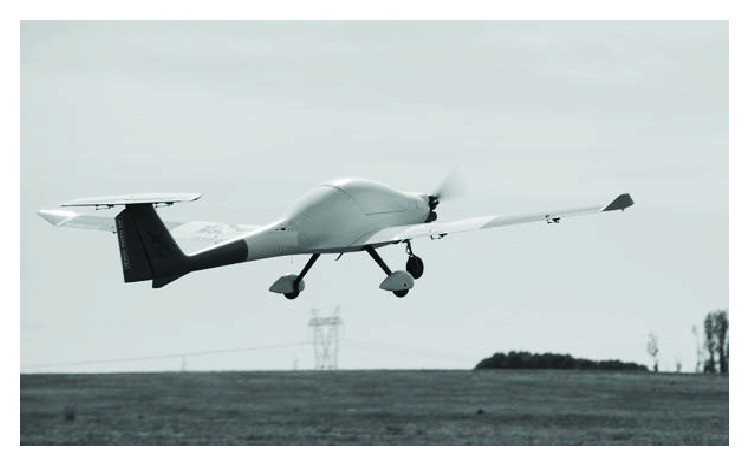
KUAV during test cases.

Figure 19 shows the onboard equipment mounted on the UAV. In Figure 20, the UAV during the test cases is shown.

## 7. Conclusions

The dynamics of the UAVs are highly nonlinear and continuously vary with time. Also, it is subjected to severe external disturbances. Due to this, dynamic and parametric uncertainties arise in the mathematical model of the UAVs over different operating conditions. This paper addresses the problem of designing a robust control system for UAVs in the presence of uncertainties using *H*_*∞*_ technique. The controller implemented allows the UAV to track all types of manoeuvres in the presence of noisy environment. The reference vector used, formed by vertical velocity, true airspeed, and heading angle, suggests a nontraditional way to pilot the aircraft that is based on commanding the desired reference vector and lets the controller select throttle position and surfaces deflections. This kind of pilot-machine interaction appears to be a more intuitive approximation.

The frequency domain analyses show that the proposed controller guarantees good performance, attenuating high frequency noise and also supplying suitable control signals. The tracking performance of the UAV is within the desired tracking performance range. The control efforts during soft manoeuvres are in the same way moderated. The first results obtained with the real UAV with the controller designed appeared to be very suitable.

The desired behaviour is introduced using a matching model (*M*). This architecture allows modifying the desired performances without varying the controller architecture.

The architecture selected to decouple the longitudinal and lateral dynamics provides very good performances. The outer-loop controller gives a very good behaviour in case of step responses, ramp responses, and combinations of these two input types. It is important to note that the outer loop shows a signal derivative at the input and this should be avoided. This signal derivative is not part of the real implementation. In this case, the inputs of the outer loop are provided directly by the GPS (height and vertical velocity). The specifications and robustness performances have been validated by mean of simulation and real tests.
